# Compliant robotic behaviors for satellite servicing

**DOI:** 10.3389/frobt.2023.1124207

**Published:** 2023-07-18

**Authors:** Joseph Cressman, Rahul Pokharna, Wyatt Newman

**Affiliations:** Department of Electrical, Computer, and Systems Engineering, Case Western Reserve University, Cleveland, OH, United States

**Keywords:** compliance, admittance control, remote supervision, teleoperation, behaviors, satellite servicing Frontiers

## Abstract

The demands of traditional industrial robotics differ significantly from those of space robotics. While industry requires robots that can perform repetitive tasks with precision and speed, the space environment needs robots to cope with uncertainties, dynamics, and communication delays or interruptions, similar to human astronauts. These demands make a well-suited application for compliant robotics and behavior-based programming. Pose Target Wrench Limiting (PTWL) is a compliant behavior paradigm developed specifically to meet these demands. PTWL controls a robot by moving a virtual attractor to a target pose. The attractor applies virtual forces, based on stiffness and damping presets, to an underlying admittance controller. Guided by virtual forces, the robot will follow the attractor until safety conditions are violated or success criteria are met. We tested PTWL on a variety of quasi-static tasks that may be useful for future space operations. Our results demonstrate that PTWL is an extremely powerful tool. It makes teleoperation easy and safe for a wide range of quasi-static tasks. It also facilitates the creation of semi-autonomous state machines that can reliably complete complex tasks with minimal human intervention.

## 1 Introduction

Space operations have long been a driver of robotic technology. Maintenance tasks often require complex manipulation and real-time adaptability at which humans excel. Unfortunately, these tasks also expose astronauts to dangerous conditions and waste their extremely valuable time. Ideally, robots should perform dangerous tasks under the supervision of ground based operators. In practice, transmission latency and interruptions make this extremely difficult. A robust and adaptable control scheme is necessary to overcome these challenges.

This paper considers the premise of a space robot under supervisory control from Earth performing necessary but challenging tasks. These can include: stowing and acquiring tools, performing peg-in-hole (or sleeve-on-peg) mating operations, performing snap-fit operations, capturing a floating object, berthing the captured object, manipulating hinged doors/panels, inserting plugs or mating connectors, and assembling cover plates and other extended objects. In this study, it is assumed that operator commands and feedback from the remote robot would experience potentially large latency. Our intent was to evaluate the use of “soft attractors” as a means of implementing robot behaviors applicable to the above operations. Further, the supervisory control interface available to the operator should be simple, natural and effective.

In terrestrial applications, safety, efficiency and competence can often be synergistically achieved by pairing a human operator with a robot. Competence can generally be increased by closely coupling the remote task to the human operator’s actions and senses. For example, in First Person View (FPV) drone racing, an operator wears goggles that stream live video from the drone directly to the eyes. This helps to immerse the operator; making them feel like they are actually piloting from a cockpit on the drone. Combined with the analog joysticks on the remote controller, FPV allows the operator to react quickly to obstacles and to naturally adjust to variable wind conditions (DJI, 2022).

Unfortunately, for many situations immersive telepresence is impractical or even counterproductive. If latency between performing an action and observing the result is on the order of 1/10th of a second, the operator may experience nausea Stauffert et al. (2020). Significant time delays also have the potential to cause instability Currie and Peacock (2002). When an operator performs an action and does not immediately see results, they may exaggerate their input to compensate for perceived unresponsiveness. Experienced operators can usually learn to adapt to substantial delays, but this comes at a cost of speed and efficiency. With large delays, operators are forced to break up a task into a set of small motions separated by pauses. Task completion time increases linearly with latency Ferrell (1965).

To address these problems, Ferrell and Sheridan (1967) introduced supervisory control. Instead of relying on the reactions and motor skills of a human operator for real-time control, the remote robot operates with relative autonomy at the lowest level and responds to higher-level directives from the human “supervisor”. For robustness, the remote computer should be able to deal safely with any contingency that may arise without human intervention. This may mean that the remote computer only handles relatively short movements. For efficiency and speed, however, the human operator should interfere as infrequently as possible. For a given task and latency, a relative sweet spot exists that balances the level of human control with autonomy. With a more robust and independent autonomous subsystem, less human intervention is required and the task can be completed more quickly.

Even in terrestrial applications with little to no transmission latency, supervisory control can be extremely useful. Immersive telepresence systems are expensive and complex, and supervisory control greatly facilitates manipulation tasks that would otherwise require extremely complicated and precise commands. For example, researchers in the 2015 DARPA robotics challenge found supervisory control to be essential for manipulation tasks. For known manipulation tasks, such as turning a valve (Newman et al., 2014) and cutting through a wall (Chong et al., 2015), it is more reliable and faster for an operator to initiate predefined skills rather than direct fine motor movements.

In order to achieve robustness, the robot should be able to respond to its circumstances in real time. One of the most important events is contact. If the robot makes contact with something in its environment and fails to adjust its motion in response, it can very easily damage itself or its environment. One of the simplest methods by which a robot can respond to contact is the “guarded move.” Under a guarded move, a robot carries out a motion until contact forces exceed some threshold, at which point motion is terminated. Many industrial robots have this kind of functionality built into their controllers at a very low level, and will shut down if actuator currents get too high. While the guarded move is very safe, on its own it does very little to continue the task after contact.

A more effective approach is to dynamically adjust the motion in response to forces. This is superficially similar to the way humans use tactility to accomplish manipulation tasks (Flash and Hogan, 1985; Rosenbaum, 2010; Enoka and Duchateau, 2017). When robots do this, it generally falls under the umbrella of compliant motion control (also called “force control”).

Considerable research has been devoted over the last 50 years to designing compliant-motion controllers with force feedback. Challenges in designing such control include latency, robot link and transmission flexibility, and servo controller bandwidth. These problems are exacerbated in space robots, since they typically must be lighter (and thus more flexible), and space-rated computing components are less powerful than domestic electronic components (thus exacerbating controller bandwidth limitations).

While these are significant challenges, they are not addressed here. Rather, this presentation starts with the assumption of a viable compliant-motion controller and instead focuses on the next layer of abstraction–manipulation behaviors via supervisory control of a compliantly-controlled robot. In our experiments, we used a form of “admittance control” (similar to impedance control). Admittance control is a form of compliant motion control in which the robot is driven to behave with a defined mechanical admittance (the reciprocal of mechanical impedance). Ideally, an admittance controller should maximize the motion response to an applied force while also maintaining stability. This is especially useful for human-robot interaction because it allows the robot to be easily controlled by applied forces Keemink et al. (2018). The controller can be designed to passively interact with the environment, which guarantees stability Dohring and Newman (2003). This is extremely useful for space applications, since instability could result in irrecoverable damage to equipment in orbit. Importantly, the use of an admittance controller accommodates implementation of virtual attractors and virtual wrenches, on which the present study depends.

Use of compliant-motion control and soft attractors offers opportunity for safe and gentle interactions. Given such an underlying system, the next layer of abstraction to consider is, how should the compliance parameters (stiffnesses and dampings) and soft attractor trajectories be generated to perform useful tasks? To help address this challenge, three parameterized “behaviors” are presented, and these behaviors are shown to be effective in performing a wide variety of interaction tasks. These behaviors can be invoked incrementally under supervisory control. Additionally, they constitute effective building blocks for constructing state machines that achieve higher levels of autonomy.

Behaviors have been employed for assembly tasks in the past to great effect. Behaviors built on top of compliant motion controllers have been especially successful (Newman et al., 2006). In our research, we distilled a variety of compliant behaviors into a much smaller yet still extremely powerful set of behaviors.

## 2 Methods

### 2.1 Overview

**FIGURE 1:**
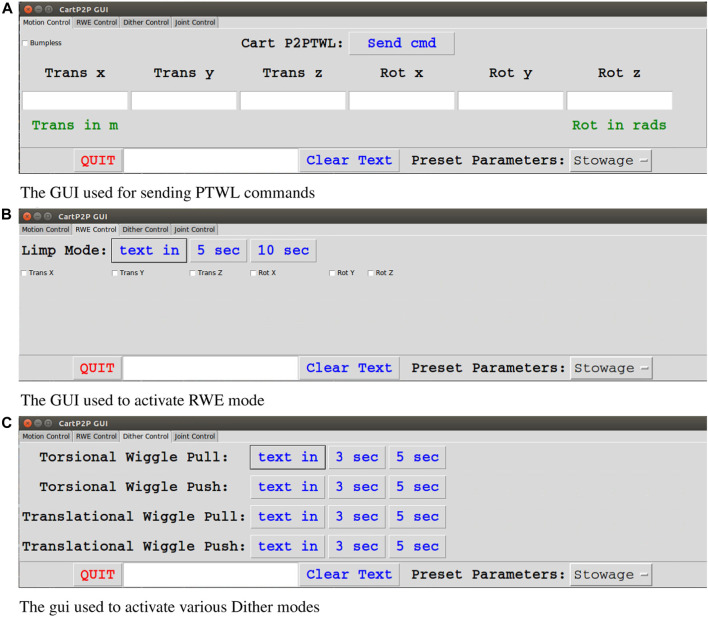
The various tabs of the GUI used to control the robot. A dropdown at the bottom right allows the selection of predefined stiffness/damping profiles. **(A)** The user enters the attractor translation and rotation and hits “Send cmd” to activate a PTWL move. **(B)** The user can choose which axes and how long to reset equilibrium. **(C)** The user can select between 4 dither presets.

**FIGURE 2:**
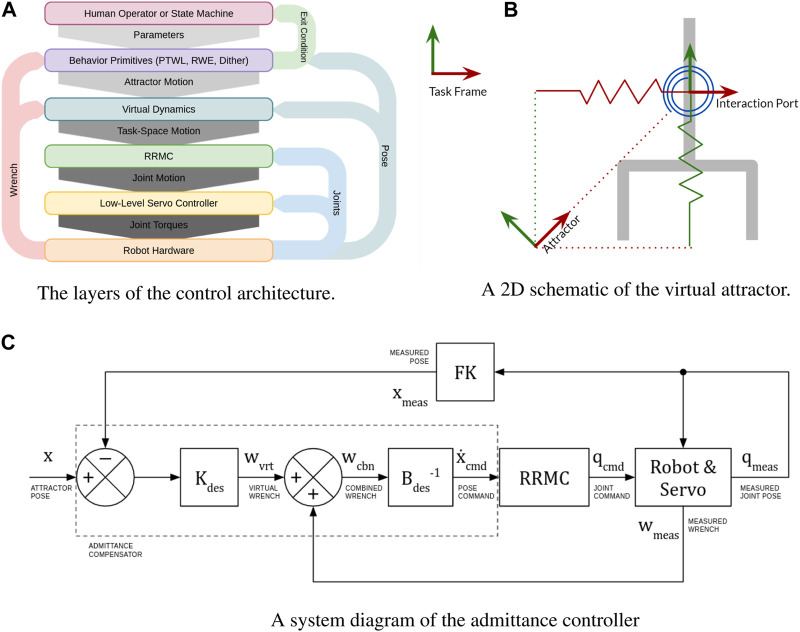
An overview of the robot’s control layers. **(A)** Layers of abstraction with the user at the top and the hardware at the bottom. Arrows represent information flow. **(B)** The attractor is attached to the port of interaction with virtual springs. **(C)** The admittance compensator generates a task-space velocity that balances virtual damping forces with virtual elastic forces and measured contact forces.

The control architecture for our robot is comprised of several layers of increasing abstraction. At the lowest layer, the robot joint velocities are controlled by a high-speed servo loop. The joint velocities are specified by an admittance controller at the next layer. The admittance controller receives force input from a sensor in series with the robot’s tool flange and virtual forces computed from the virtual attractor. The pose of this virtual attractor is controlled by various behavior primitives that move the attractor in a manner defined by a handful of parameters. Once a force threshold is crossed or a desired pose is achieved, the behavior terminates and returns an exit condition. At the highest layer, a human operator or an automated state machine selects behavior primitives and appropriate parameters. hlThe operator selects between these behavior primitives using a graphical user interface (GUI) with a mouse and keyboard. While joysticks or other analog motion controls are useful in a live environment, the robust nature of our behaviors means that comparatively slow, point-and-click controls are perfectly sufficient for compliant supervisory control. A screenshot of the user interface can be seen in Figure 1. In the case of a state machine, the subsequent state depends on the exit condition returned by the behavior. Behavior primitives are executed sequentially until the task is completed. This architecture is summarized in Figure 2A.

#### 2.1.1 Admittance control and virtual attractor

The goal of the admittance controller is to “admit” environmental forces via its virtual dynamics. In our implementation, this is achieved through the use of a “Virtual Attractor.”

The concept of virtual attractors was championed by Hogan, based on theories and experiments in sensorimotor control in primates (See, e.g., Hodgson and Hogan (2000), Hodgson and Hogan (1992)). This work inspired researchers to use virtual attractors for robot controls in the context of mechanical assembly and force-sensitive interactive tasks (See e.g., Newman et al. 124 (2003)).

The attractor consists of a desired pose together with a matrix of virtual stiffness. The attractor is attached to the robot via “virtual springs” at the “port of interaction,” which is most conveniently the frame of the force-torque sensor, as illustrated in Figure 2B. Together, these are used to generate virtual forces that compete with the forces measured by the force-torque sensor.

While there are many admittance-control implementation variations, the method used for the experiments reported below as follows. It is heavily related to the controller described by Maples and Becker (1986) and passivity analysis described by Balajepalli (2020).

$$0=w_{\mathrm{net}}=w_{ft}+{\tilde{w}}_{ft,K}+{\tilde{w}}_{ft,B}$$ (1)

$${\tilde{w}}_{ft,B}=-B_{\mathrm{des}}t_{ft}$$ (2)

$$0=w_{ft}+{\tilde{w}}_{ft,K}-B_{\mathrm{des}}t_{ft}$$ (3)

$$B_{\mathrm{des}}t_{ft}=w_{ft}+{\tilde{w}}_{ft,K}$$ (4)

$$t_{ft,des}=B_{\mathrm{des}}^{-1}\left[w_{ft}+{\tilde{w}}_{ft,K}\right]$$ (5)

In the above, **w**_*ft*_ is the force/torque wrench exerted by the environment on the interaction port of the robot. This is assumed to be identical to the force/torque that is measured at this point. The terms ${\tilde{w}}_{ft,K}$ and ${\tilde{w}}_{ft,B}$ are virtual wrenches, computed based on the assigned (desired) virtual stiffness and virtual damping at the interaction port. The virtual wrench due to the virtual spring scales with the stretch between the virtual attractor pose (defined per trajectories generated for specific tasks) and the actual pose of the interaction port (computed in real time via forward kinematics). The virtual wrench due to damping scales with the twist (6-DoF velocity) of the interaction port, **t**_*ft*_, and this quantity is also computed in real time based on the measured joint velocities and the robot’s Jacobian. The compliance controller (as implemented) attempts to achieve the twist vector **t**_*ft*,*des*_ that is consistent with the defined physical and virtual interaction wrenches. This is accomplished by continuously recomputing **t**_*ft*,*des*_ and using a Resolved Rate Motion Controller (which uses the Jacobian inverse) to convert this task-space velocity into joint motions that are sent to the robots internal servo. This subsystem is illustrated schematically inFigure 2C.

This control scheme is useful when the robot is moving unobstructed through space, and when it is performing tasks. In free-space, the robot will just follow the virtual attractor as quickly as **B**_*des*_ allows. This eliminates the need for careful second-order trajectory planning, as the damping will ensure that the robot never moves too quickly. In contact, the robot will still try to follow the position commands, but it will never allow the contact forces to get too high. When necessary, higher forces can be easily achieved by simply moving the attractor farther in the desired direction.

### 2.2 Behaviors

Regardless of the specific compliant-motion controllers implementation, the intent is to provide a foundation for performing interactive tasks in which contact wrenches must be exerted, regulated and interpreted. To exploit an underlying compliant-motion controller, a subsequent layer of abstraction may be defined, described here as compliant “behaviors.”

Behaviors are actions that a robot can perform without operator intervention. Simple behaviors can be made extremely reliable and robust, and these behaviors can be invoked directly, under supervisory control, or chained together in state machines to create higher levels of autonomy.

We have found three specific behaviors that have been remarkably capable and flexible. It will be shown that these three behaviors can be exploited to perform a wide variety of manipulation tasks while exhibiting remarkable robustness. These three behaviors are optimized and generalized versions of those described by Haberbusch (2020), and are capable of performing the same functionality with fewer steps. This simplifies both teleoperation and state-machine design.

#### 2.2.1 PTWL

PTWL is a simple scheme for generating attractor motion. PTWL is defined by a handful of parameters: 1) a desired displacement of the attractor, 2) the duration of the attractor move, 3) a force/torque threshold, 4) a pose tolerance, 5) the stiffness matrix, 6) the damping matrix, and 7) a watchdog timer. Once PTWL begins, the attractor is moved at a constant velocity toward the desired pose in the specified amount of time. This will continue until the desired pose is reached within tolerance, or the force/torque threshold is exceeded, or the watchdog timer runs out. Once any of the exit conditions are met, the attractor is immediately frozen and remains stationary until another command is given.

In general, all of these parameters are held constant for a given task, except for the desired displacement. This allows the operator to focus on plotting desired motions of the manipulator to complete the task. PTWL offers extremely intuitive control over both position and force. This allows it to be applied to a wide range of manipulation tasks that involve fine control over forces and motions.

#### 2.2.2 RWE

The behavior “RWE”, for “Reset Wrench Equilibrium”, moves the attractor to be coincident with the port of interaction. As a result, the virtual wrenches (both stiffness and damping) are definitionally set to zero, and only the sensed (physical) wrench contributes to the desired twist. The desired twist, via the compliance controller, converges to zero as the contact wrench is driven to zero. This relieves contact forces without substantially changing the robot pose. RWE is useful between PTWL moves, especially if the move terminated on a force/torque threshold. If PTWL were used by an operator or state machine without RWE, it could get stuck every time a force threshold were crossed. RWE allows the operator to easily relieve contact efforts without substantially moving the robot end-effector.

#### 2.2.3 Dither

Over the course of several experiments, some sequences of attractor movements tend to appear multiple times in various contexts. These sequences offer enough utility to warrant their own separate behavior. One example is “dithering”, in which the attractor oscillates while simultaneously moving in a specified direction, usually in order to free itself from a stuck position. Dithering was found to be especially useful in the tight-tolerance sleeve-on-peg task, described below, where any misalignment could cause the sleeve to become lodged in place. In order to dislodge, the sleeve needs to be moved in a specific direction that depends on the exact way in which it is lodged. RWE is often insufficient for this because it will only move the attractor until forces are reduced, which does not necessarily solve the problem. Dithering, however, wiggles the attractor in all directions until the correct one is found by trial and error.

Several dither modes were created to deal with various circumstances in which they were found useful. These are divided into torsional and translational dither modes. In torsional mode, the attractor rotates about a specified axis while keeping its origin on the axis. In translational mode, the attractor orientation is fixed while its origin is dragged in circles about the specified axis. These two modes are divided further into push and pull modes, where the attractor is either moved forward or backward along the specified axis. As with the other behavior primitives, dither modes can be activated at the discretion of a human operator or can be triggered by a specific contact event in a state machine.

### 2.3 Tasks

One potential application of space robotics is to extend the life of existing satellites by capturing and refueling them. This operation requires several distinct phases. First, during the capture phase the robot must grapple the satellite. Grappling is dominated by dynamic interactions, and also requires machine vision or some other form of tracking Strube et al. (2012). Admittance control has been proposed and tested as an appropriate control scheme for robotic satellite capture Wu et al. (2017). The efficacy of our controller for capture is currently being experimentally tested with air bearings in a 3-DoF (x-y translation and z rotation) emulation of zero gravity.

After the satellite is captured, it must be berthed. This involves manipulating the satellite into position such that it can be held in place by fixed posts on the servicing vehicle. Once successfully berthed, the servicing phase can begin. This may involve several servicing tasks, such as cutting, refueling, and tool exchange.

For each of these tasks, a terrestrial analog was created in the lab environment to roughly simulate the geometry and forces involved. This is an effective testing method, despite the inherent differences between the laboratory and space environments and is in many ways superior to purely virtual simulation (Carignan et al., 2014). The tasks were carried out with the terrestrial analog to determine appropriate parameters and control strategies. These analogs were all 3-D printed using PLA and/or constructed using commonly available materials. For those interested in replicating setups, CAD files for 3-D printed parts can be found at (Nguyen, 2021). The robot used for experiments was an IRB120 manufactured by ABB.

Other space robotics applications involve a variety of as-of-yet unforeseen operations. It is anticipated that PTWL and the compliant behaviors developed here are sufficiently versatile to address almost any quasi-static manipulation task. To demonstrate this, several tasks that may be useful for servicing, assembly or manufacturing were tested.

#### 2.3.1 Berthing

As discussed above, berthing involves manipulating the satellite into a specific position so that it can be held in place for servicing. For terrestrial experiments, the 6-degree-of-freedom satellite is modeled using 3-DoF planar air bearings. The bearings are fixed to an elongated sled and weights are distributed such that the moment of inertia is large and the center of gravity is significantly offset from the port of interaction, as would likely be the case for a robot externally gripping a large satellite bus.

The air bearings were constructed from scratch using 3-D printed shells and porous graphite pucks in similar fashion to (Preiss, 2019), and are powered by a 60psi compressed nitrogen tank. The sled and grip were constructed from aluminum and wood using hand tools.

**FIGURE 3:**
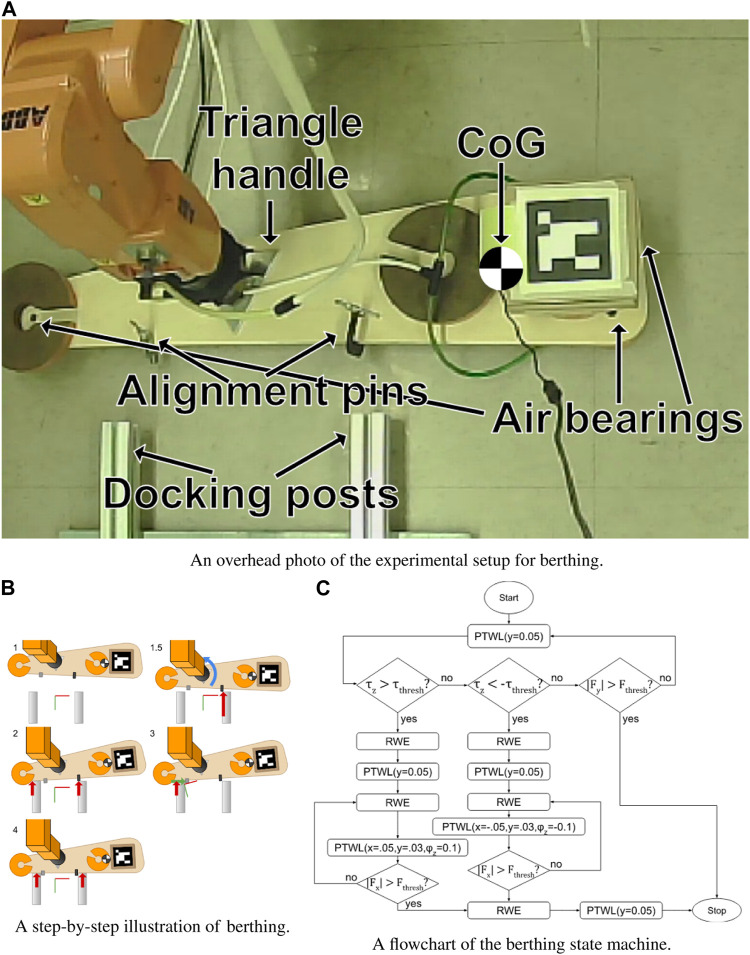
An overview of the berthing process. **(A)** The satellite analog is fixed to the robot’s end-effector, and has two protrusions that mate with the inside of the posts. A video example of manually supervised berthing can be seen at Cressman (2022a). **(B)** The thick arrows represent contact forces. The blue arrow in step 1.5 illustrates the torque felt at the robot end effector that is used by the state machine to determine which side made first contact. **(C)** Rhombuses denote decisions and rectangles denote attractor moves. A video example of the state machine in action can be seen at Cressman (2022c).

In the 3-DoF emulation, the payload is to be manipulated to align with two posts. The robot grips a handle on the payload using a pneumatic three-jaw chuck, which constrains all three degrees of freedom of the payload. These components are labeled in Figure 3A. Additionally, the virtual stiffnesses of the attractor are set to near zero in all degrees of freedom constrained by the plane (*z*, *ϕ*_*x*_ and *ϕ*_*y*_). This mitigates issues that could arise from slight misalignment of the robot base from the payload (emulated satellite) plane, such as lifting or tilting.

Experimentally, the robot was controlled under incremental supervisory commands to achieve berthing, thus demonstrating that berthing is completely achievable through the previously developed compliant behaviors, PTWL and RWE. This process is illustrated in Figure 3C (The red-green axes represent the location of the virtual attractor). With the satellite roughly aligned with the berthing posts 1), the virtual attractor was moved toward and past the posts until the satellite contacted and pressed against both of them 2). Since the original alignment was imperfect, this step usually left the satellite canted, with one clamp touching one post and the other post just touching the body of the satellite 2). Looking at the camera view, one could see which post was in contact, and in which direction to move the satellite to correct the alignment. To make this correction, RWE was first exerted to eliminate contact forces. Subsequently, the attractor was displaced to induce sliding sideways in the appropriate direction while still maintaining forward pressure 3). Finally, once the second post made contact with the second clamp, the attractor was displaced forward (in the positive *y* direction), drawing the satellite into its berthing pose, satisfying multiple physical constraints.

After the task was proven possible under supervisory control, a state machine was designed to carry out the task autonomously. The process used by the state machine is very similar to that used by the human operator, but modified slightly for a sole reliance on force feedback with no supplementation by visual cues. Specifically, the system needs to determine whether the payload is aligned with the posts and, if not, in which direction it needs to move to correct the alignment.

This determination can be made using torque measurements at step 1.5 in Figure 3B. If the satellite is misaligned when first contact occurs, then the robot will measure a substantial torque. If this torque is positive, then it needs to move in the positive-x direction to correct the alignment. Conversely, if the torque is negative (as in the figure), then the payload will need to move in the negative-x direction (to the right in the figure).

After measuring this torque, the robot can continue to press forward until both posts are pressed against the satellite (Step 2). Next, the robot slides the satellite in the direction that was determined by the torque measurement in step 1.5. At the same time, it continues to apply forward pressure, as well as a slight torque in order to maintain contact with both posts. Once the ring contacts the second post (Step 3), the satellite is horizontally aligned and the berthing can be completed by simply pressing forward. Figure 3C is a flowchart summarizing this algorithm. In the flowchart, *τ*_*z*_ is the torque about the *z* axis, *F*_*x*_ is the force along the *x* axis, *F*_*thresh*_ is the contact force threshold parameter, (15N for these experiments) and *τ*_*thresh*_ is the contact torque threshold. *PTWL*(*x*, *y*, *z*, *ϕ*_*x*_, *ϕ*_*y*_, *ϕ*_*z*_) displaces the virtual attractor from the port of interaction by the specified dimensions. RWE moves the attractor back onto the port of interaction to eliminate contact forces. This state machine was able to successfully and consistently berth the satellite even in the presence of significant perturbations.

#### 2.3.2 Tool exchange

**FIGURE 4:**
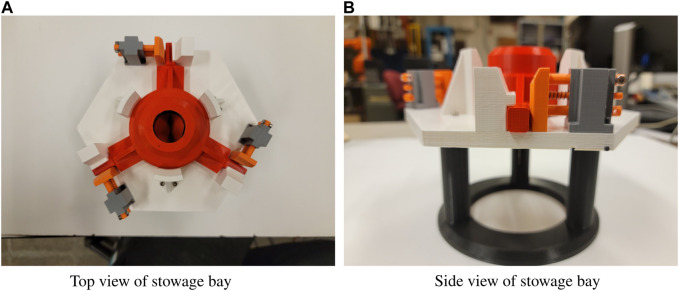
The tool stowage bay is the most complex task with the longest series of required commands to successfully complete the task. A demonstration of tool stowage can be seen at Pokharna (2021d) and of tool retrieval at Pokharna (2021c).

The stowage bay (seen in Figure 4) is an example device for tool changes. This bay was designed in SolidWorks by Quan Nguyen and 3D printed from PLA. Multiple bays may be used to store additional tools, which can then be retrieved and stowed by the robot. The stowage bay has a set of three locks which are held shut by springs. Each lock holds a leg of the tool to secure it in place. In order to stow the tool, the robot unlocks the bay, bottoms out the tool within its compartment, and then locks the bay once more before releasing the tool. For retrieval, the bay must be unlocked and then the tool removed from the bay. A demonstration of stowage can be seen at Pokharna (2021d) and retrieval at Pokharna (2021c).

This task has elements where the wrench limiting of PTWL will assist in determining whether or not the bay is fully unlocked. The use of compliance assists with the alignment of the tool, ensuring proper orientation and fit within the bay.

The PTWL and RWE behaviors were found to be adequate for supervisory control of tool changes. Importantly, these behaviors were found to be suitable for constructing a state machine for autonomous tool changes. Since tool changes can be a frequent operation, autonomous tool changing is particularly attractive. Using this state machine, the process of retrieving and stowing the tool can be done with the press of a button. This automation was used to successfully complete 100 consecutive trials of both retrieval and stowage without a single failure, proving the robustness and reliability of the state machine and the behaviors it was built from (Pokharna, 2022).

#### 2.3.3 Tool retrieval

Completing a tool retrieval requires a few steps. First, the robot must bottom its contact with the tool before engaging the gripper to grab the tool. Once the tool has been secured, the stowage bay lock must be rotated open before extracting the tool. The task is complete once the tool is removed from the stowage bay. Following these steps, an operator achieved success in every trial run, including cases with long communications latency.

**FIGURE 5:**
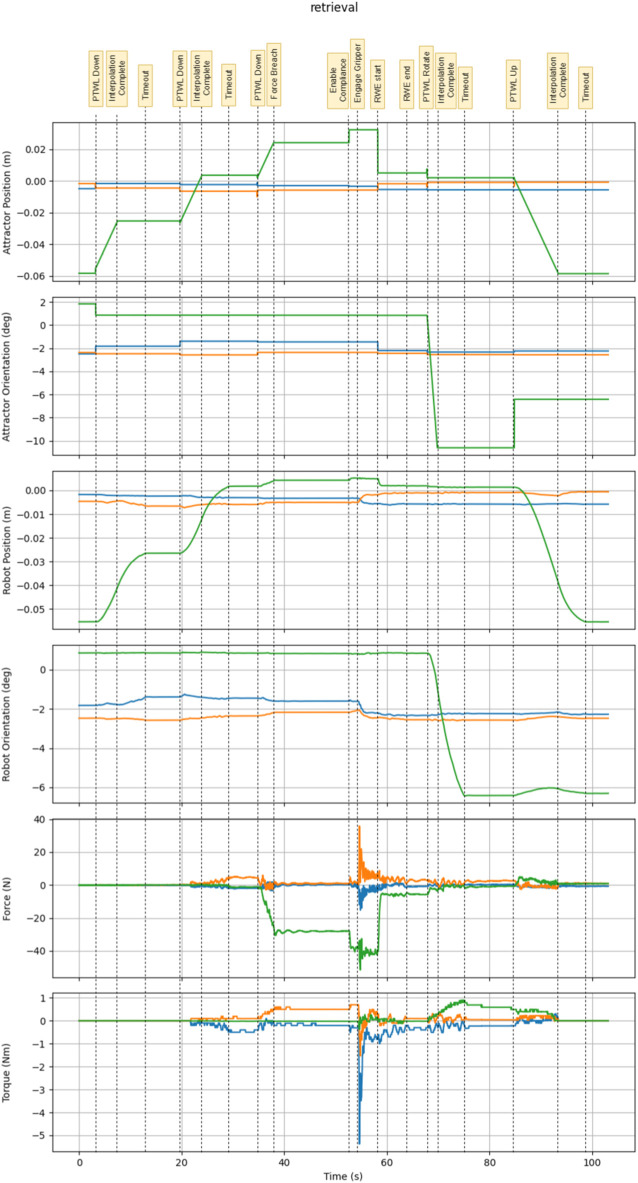
This is an annotated graph showing the state of the system during the retrieval task.

An annotated example of tool retrieval can be seen in Figure 5, which labels each transition point on the graphs. The test results can be seen in Table 1 below.

**TABLE 1:** The list of behaviors used and wrench statistics for all trials of tool retrieval.

Comparison of wrench metrics		
	Retrieval	Retrieval Delayed
Mean Max Force	51.829	20.751
Mean Max Torque	5.296	2.549
Mean Run time	99.761	93.144
Mean Num Behaviors	6.2	3.6
Number of Trials	10	10
Combined behavior breakdown		
Total Number RWE	10	6
Total Percent RWE	16.13%	16.67%
Total Number PTWL	52	30
Total Percent PTWL	83.87%	83.33%
PTWL Timeout	42	29
PTWL Goal Reached	0	0
PTWL Wrench Breach	10	1
Total Num Behaviors	52	36

Some relatively large forces and torques can be seen during tool retrieval. This are induced by the impact of the pneumatic gripper when grabbing the tool stored within the stowage bay. The gripper was connected to an air supply with 70psi, and gripper actuation was rapid. Due to imperfect alignment between the gripper and payload, large forces/torques could be generated rapidly upon gripper actuation. With the robot under position control, these efforts could be large and sustained. Under compliant motion control, the robot inherently performed fine adjustments to reduce the contact forces. After the gripper is engaged, the RWE behavior is used to finely adjust the attractor position and further eliminate forces.

Since the tool stowage device was at a known, repeatable pose with respect to the robot base, the robot could be commanded to a fairly precise grasp pose using position control. However, even a precise approach was insufficient to assure low interaction force when the gripper was actuated (thus forming a closed-chain constraint). To address this, the robot was first sent to a precise grasp pose, then its controller was switched to compliant motion prior to engaging the gripper. This transition from position control to compliant-motion control was done in a “bumpless” fashion by setting the attractor pose equal to the robot’s pose. (Bumpless transition was also employed when the robot was in contact with non-zero forces, which required computation of an initial attractor displacement away from the robot’s current pose).

By enabling compliance before engaging the gripper, the system was able to react and adjust to some of the loads, with any remaining loads reduced with an RWE once the tool was secured. Through experimentation, this series of commands greatly reduced the frequency of emergency shutdowns of the robot due to excessively high loads, as well as reduced the maximum loads experienced by the system.

Under supervisory control, PTWL was found to be the most frequently used behavior for tool retrieval trials. RWE was invoked when behaviors terminated due to a wrench breach.

The features of PTWL allowed large commands to be issued safely, reducing the total number of behaviors required to complete a tool retrieval. When moving the robot into contact with the tool from the approach pose, a large virtual attractor displacement command would guarantee contact while the wrench limit would ensure the loads were within the operation limits. The same applied to rotation to unlock the stowage bay. Operators became more proficient with this task over time, and observations of skilled humans using these behaviors informed the design of autonomous state machines for tool changes.

#### 2.3.4 Tool stowage

**FIGURE 6:**
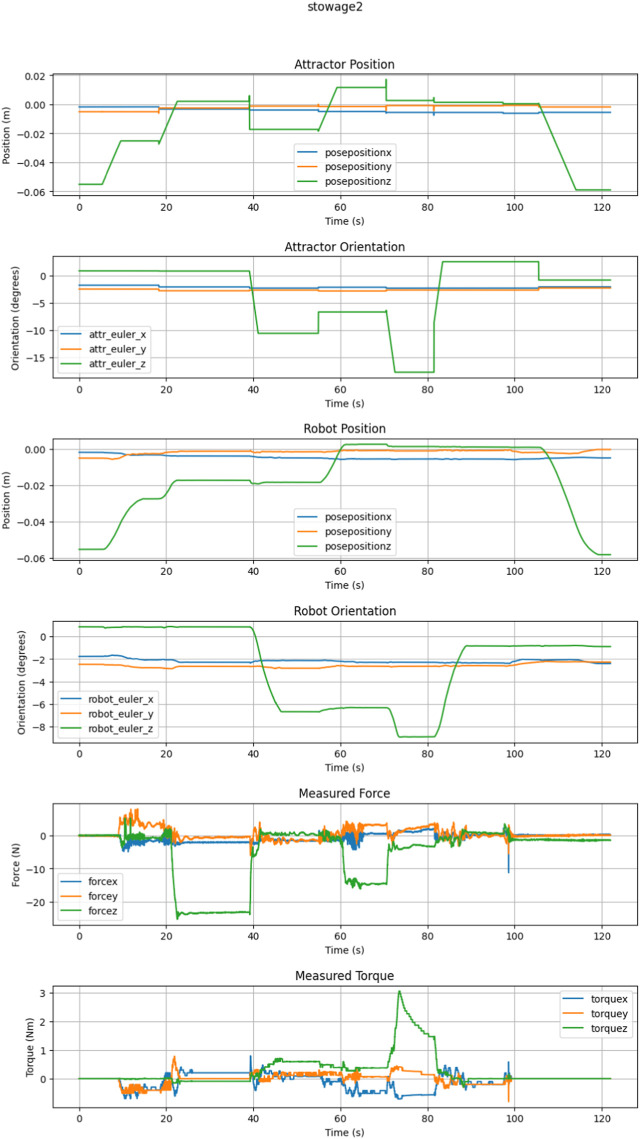
This is an example trial graph showing the state of the system during stowage.

The tool stowage task is more complex than tool retrieval as it requires more commands to complete the task. Tool stowage requires coming into contact with the upper stop of the stowage bay, rotating the lock open, bottoming out within the stowage bay, and then stowing the tool by rotating the lock closed. The gripper then releases the tool before leaving the bay area. The task is completed when the tool is fully stowed within the stowage bay and the locks are all securely closed. Each trial was successfully completed under supervisory control using PTWL and RWE, with an example trial seen in Figure 6. The results of these trials can be seen in Table 2 below.

**TABLE 2:** All behaviors used during Tool Stowage.

Comparison of wrench metrics		
	Stowage	Stowage Delayed
Mean Max Force	27.28	30.03
Mean Max Torque	1.947	1.422
Mean Run time	108.762	102.112
Mean Num Behaviors	6.1	5.3
Number of Trials	10	10
Combined behavior breakdown		
Total Number RWE	0	8
Total Percent RWE	0%	15.09%
Total Number PTWL	61	45
Total Percent PTWL	100%	84.91%
PTWL Timeout	50	35
PTWL Goal Reached	1	0
PTWL Wrench Breach	10	10
Total Num Behaviors	61	53

The trials of Tool Stowage were done in conjunction with the collection of Tool Retrieval, as these tasks are complimentary. Compared to the counterpart task of tool retrieval, the max forces and torques during tool stowage were smaller. Tool stowage does not involve transient wrenches from gripper actuation. Rather, the tool is already grasped before interacting with the stowage bay.

For trials of tool stowage under supervisor control with low latency, PTWL was used exclusively. When significant latency was added, use of RWE was needed. Similar to the tool retrieval, the behaviors were able to be used consecutively and a state machine was built based on the steps that trained operators used to complete the task. The features of PTWL allowed for the fewer commands with larger motions, with a single command required for each step of the process. The behaviors were safe and reliable, with RWE rarely needed to reduce forces as the limits in PTWL kept the system within the desired operational range.

#### 2.3.5 Sleeve on peg

**FIGURE 7:**
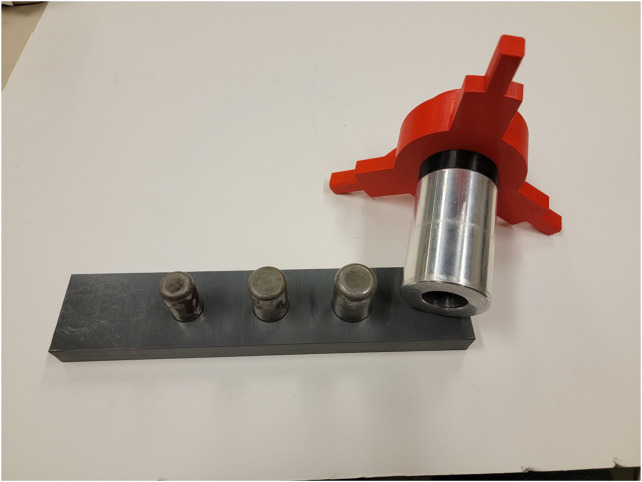
The Peg board with pegs from diameter of 0.5″, 0.75″, 1″ next to the tool attached with the sleeve outer diameter of 2″ and an inner diameter of 1.0035″. More info at Pokharna (2021b).

The simple task of fitting a sleeve over a peg is the inverse of the common peg-in-hole task. The sleeve-on-peg task adds in some additional complexity that a peg-in-hole task may not have, where something can press on the inside or the outside of the sleeve on the tool. Additionally, this task is an abstraction of other useful tasks, like fitting a tool over a bolt to remove it or attaching a nozzle over a fuel valve. This abstracted task is one that could be required frequently for a variety of tasks, and thus was created with a very tight clearance to be deliberately challenging in order to show the usefulness of the compliant behaviors. The aluminum sleeve had an outer diameter of 2″ and an inner diameter of 1.0035”. The steel peg board had a series of different pegs, at 0.5″, 0.75″, and finally the 1″ peg as seen in Figure 7. The pegs with larger clearance were easily handled with supervisory control and PTWL. The low-clearance peg presented the greatest difficulty, and thus this case was explored more fully. This clearance imposed a strict orientation constraint, as the tool was prone to jamming. The peg was mounted on a stowable tool, which added an additional layer of difficulty, since this extended the grasped sleeve further from the robot’s wrist.

**FIGURE 8:**
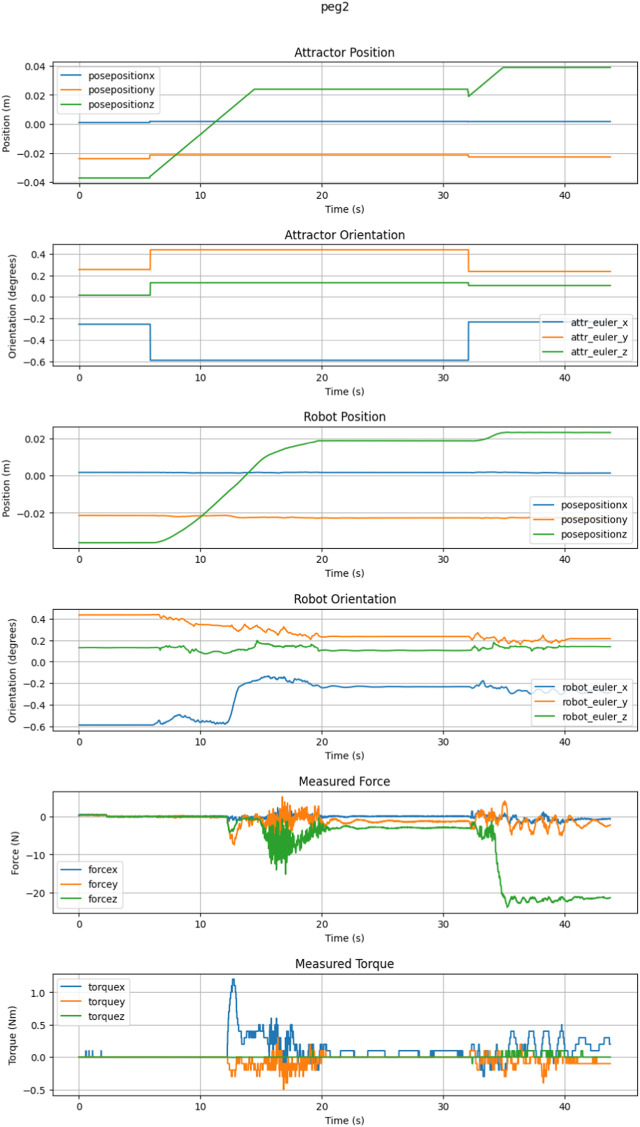
This is an example sleeve-on-peg trial requiring two commands.

The sleeve-on-peg task was the most demanding of the tasks presented here. With the tight clearances, if there was even a small misalignment of the insertion axis, the sleeve would jam on the peg. Despite this, the compliant behaviors were typically able to smoothly insert the sleeve over the peg with only a single command. An example trial can be seen in Figure 8, where only two commands were required. The results of the trials are in Table 3 below. Supervisory control using only the three described behaviors was successful both with low and high communication latency.

**TABLE 3:** The breakdown of all behaviors used during the sleeve-on-peg trials.

Comparison of wrench metrics		
	Sleeve-on-peg	Sleeve-on-peg Delayed
Mean Max Force	18.763	19.562
Mean Max Torque	1.354	1.619
Mean Run time	18.2	14.554
Mean Num Behaviors	1.2	1
Number of Trials	10	10
Combined behavior breakdown		
Total Number RWE	0	0
Total Percent RWE	0%	0%
Total Number PTWL	12	10
Total Percent PTWL	100%	100%
PTWL Timeout	12	10
PTWL Goal Reached	0	0
PTWL Wrench Breach	0	0
Total Num Behaviors	12	10

When attempting an insertion with an intentional misalignment, the sleeve itself would regularly become jammed and would breach a wrench limit with each PTWL. When using an RWE, the loads would be reduced, but jamming would still occur after a PTWL was sent. This happened regardless of the new commanded pose. In these cases, the dithers were useful to either insert further or retract and attempt to reinsert with a new alignment.

A variation of RWE was found to be useful: some interaction efforts could be preserved while the remaining components were extinguished. This variation could be used, e.g., to maintain an insertion force while relieving side loads.

#### 2.3.6 Quick disconnect coupling

**FIGURE 9:**
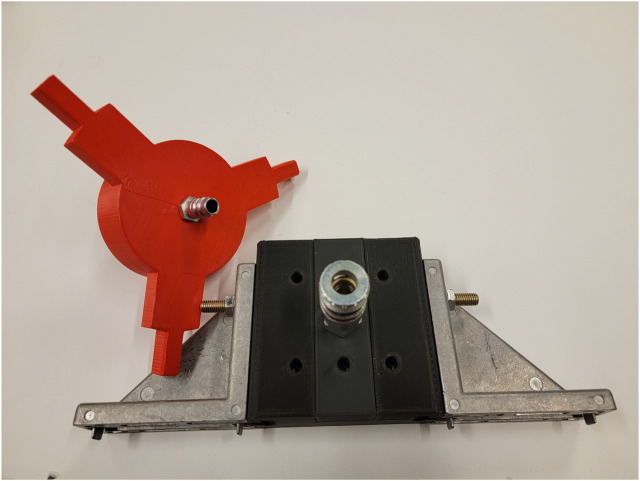
The tool with the male quick-connect adaptor next to the female connector. These are standard parts that can be ordered online, and require only a single hand for insertion. A demonstration can be seen at Pokharna (2021a).

In this task, a pneumatic quick-connect coupling operation (a snap-fit insertion) was performed. Inserting the quick-connect coupling has some properties that are similar to a regular peg-in-hole task, with the added complexity of requiring a minimum amount of insertion force. This example coupling was a conventional device commonly used in pneumatic and hydraulic systems. By mounting one of the two coupling components, this task could be completed manually with a single hand, or robotically with a single robot arm. The male part, grasped by the robot, had to be inserted into the receptacle with a minimum force between 25 N and 30 N to properly connect. These parts may be seen in Figure 9. This imposed an orientation constraint and an insertion force requirement to connect the parts.

With sufficiently precise initial alignment, a single PTWL command was typically successful in performing the task. When attempting an insertion with an intentional misalignment, the sleeve itself would regularly become jammed and would breach a wrench limit with each PTWL. When using an RWE, the loads would be reduced, but jamming would still occur after a PTWL was sent. This happened regardless of the new commanded pose. In these cases, dithers were necessary to either insert further or retract and attempt to reinsert with a new alignment. RWE with insertion force preserved was also found to be helpful.

#### 2.3.7 Plug insertion

This task involved inserting a plug into a standard US electrical outlet under supervisory control. This example is representative of a common need for performing connections, and it also is conveniently familiar as a manual operation in terms of alignments and efforts required. It is also relatively easy for a robot under position control if the exact location of the outlet is known. Without this information, however, it is very easy for the robot to miss the outlet and generate large contact forces. These contact forces can be substantially reduced using supervisory control with underlying compliant-motion control. First, the operator roughly aligns the plug with the outlet, and moves toward the surface. Once contact has been made, the operator can clearly see the direction of the offset toward the hole. The plug is moved in roughly this direction while maintaining downward force until the hole is reached, at which point the plug can be inserted.

It was found that the behaviors presented here were sufficient to perform this task under supervisory control using soft attractors. First, the attractor is placed somewhere below the outlet surface until contact is detected. Next the attractor is moved to only slightly below the surface (to maintain moderate contact force) and translated in the direction of the outlet until the force in the *y*-direction crosses the contact threshold. Then the attractor can be placed deeply into the socket, tugging the plug into engagement.

**FIGURE 10:**
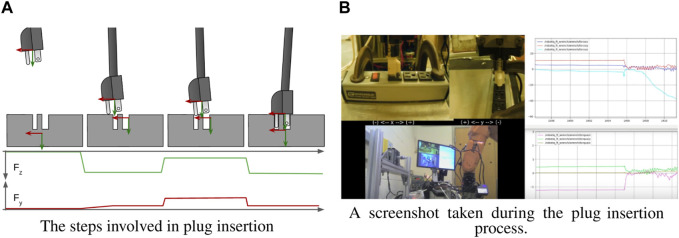
Inserting a plug into a standard US 3-prong outlet. A video of this operation can be found at Cressman (2021a).

Figure 10A illustrates the steps for plug insertion. The upper axes represent the port of interaction and lower axes are the attractor pose. This process is extremely forgiving of both spatial inaccuracies and temporal imprecision, since the different phases of the task are separated by discrete contact events. Figure 10B shows a screen capture taken during the plug insertion process.

#### 2.3.8 Opening and closing a latched door

For this task, a small door analog was fabricated, consisting of a cabinet hinge, plywood frame, door, and latch. To open the door, the robot must grab the door handle, turn the handle to unlatch the door, then translate and rotate through an arc centered around the door hinge axis. Closing the door is similar, but in reverse. This task is representative of kinematically-constrained manipulation tasks.

Just like the plug, this task is possible under strict position control, as long as the geometry of the hinge and latch are known precisely. This task would additionally require the generation of precise non-linear trajectories, specifically gripper motion about a precise 6-DoF circular arc in space. Any inaccuracies would cause the forces/torques of interaction to become excessive. Under compliance, the task is dramatically simplified and intuitive. The robot operator must only know the approximate orientation of the door handle and the door hinge. The handle can be turned by imposing a rotational displacement of the soft attractor. Subsequently, the door can be opened by setting all virtual translational stiffnesses to near zero, then imposing an attractor rotation roughly parallel to the door hinge.

**FIGURE 11:**
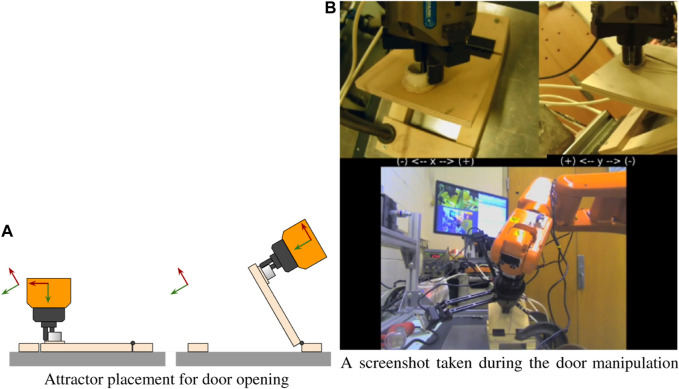
The attractor is largely translationally decoupled from the interaction port, allowing the robot to impart pure moments about the hinge. A video of the door task can be found at Cressman (2021c).

Figure 11A illustrates this strategy. The left axes in each step are the attractor and the one on the robot is the port of interaction. Since translational stiffness was nearly zero, the robot accommodated with whatever translation was necessary to minimize the interaction efforts. Consequently, the robot conformed to the hinge’s kinematic constraint while opening the door.

A snapshot of the robot performing the door task is shown in Figure 11B.

#### 2.3.9 Wide, shallow lid installation

This task involved fitting a wide disk into a hole, emulated by placing a lid on a saucepan. Though similar in principle to the sleeve-on-peg task, it is fundamentally different. In the sleeve-on-peg task, the biggest challenge is achieving the correct vertical orientation to prevent jams. In the saucepan task, the horizontal position is more important.

**FIGURE 12:**
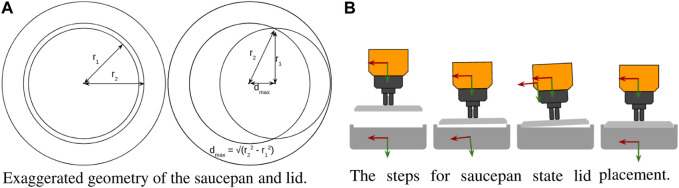
**(A)** If the initial lid placement is within *d*_*max*_ of the center, then the robot will measure an inward moment which can be used to guide the pan into place. **(B)** An illustration of the steps involved in lid installation. A video example of the saucepan task can be seen at Cressman (2021b).

In Figure 12A, the outer radius of the smaller lid *r*_1_ and the inner radius of the larger saucepan lip *r*_2_ are exaggerated to show the results of slight misalignment. In this experiment, the lid should fit inside of the lip on the saucepan. The outer radius of the lid is *r*_1_ and the inner radius of the saucepan lip is *r*_2_. If the offset *d* between the center of the lid and saucepan is less than *r*_2_-*r*_1_, then the lid can be inserted straight down without any adjustments. Otherwise, the lid will contact the lip at two points when it is pressed downward. If *d* is less than $d_{max}=\sqrt{\left(r_{22}-r_{12}\right)}$ , then a torque will be generated about the axis going through both points, causing the lid to tilt toward the center. This tilt can be measured and used to calculate the direction of the offset. If *d* is greater than *d*_*max*_, then no torque would be generated–only vertical contact forces, since the center of the lid would be on the outside of the axis through the contact points, and would be pressed flat against the lip of the pan.

A state machine was created to place the lid on the pan after it was aligned within *d*_*max*_. First, the attractor was moved down past the lip of the pan, causing the lid to contact the lip and tilt slightly. The attractor was then tilted and slid in the direction of the center of the pan until horizontal contact was detected with the opposing lip. After this contact, the attractor was reset to horizontal and pressed downward until the lid was horizontal and firmly in place. Figure 12B illustrates the steps in the lid installation process. Again, the simple behaviors presented herein were sufficient to perform this task robustly, whether under supervisory control or autonomously via a state machine.

#### 2.3.10 Socket wrench insertion

**FIGURE 13:**
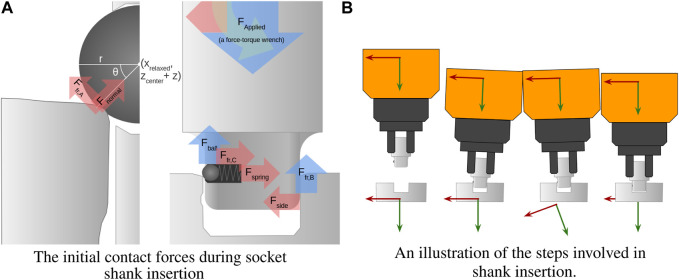
**(A)** Initially, contact forces are dominated by *F*_*ball*_, which imparts a negative moment about the port of interaction. **(B)** The attractor is rotated dramatically in the third step to depress the ball. A video example of shank insertion can be seen at Cressman (2022b).

The final task presented here used a consumer socket wrench tool-change system consisting of a shank and socket, as illustrated in Figure 13. The shank consists of a square-keyed shaft with a spring-loaded ball detent, and the socket is a mating square hole. Without the detent, the task is equivalend to a square-peg in square-hole insertion task. When inserting a peg into a hole with tight tolerances, a slight misalignment can generate contact forces that tend to amplify the original misalignment until the peg is jammed. In Pokharna (2022), it was demonstrated that a sleeve-on-peg task could be performed without any remote center of compliance by applying dithers (small horizontal perturbations) whenever a jam occurs in order to relieve friction.

Even under perfect alignment, when the shank is inserted, the ball will contact the edge of the socket. The vertical contact force dominates this interaction. Since it is offset from the axis of the applied force, a moment is induced, tilting the whole shank (clockwise in the figure) until it is jammed. Since the location of the detent on the shank is known beforehand, this jamming is predictable and can be counteracted in the same way every time, making indiscriminate dithering unnecessary.

By tilting the shank (counterclockwise in the figure), the ball detent can be depressed. Once it is depressed, the only vertical force resisting insertion comes from friction, and downward insertion becomes trivial.

Again, it was found that this operation could be performed using only the simple behaviors presented herein.

## 3 Conclusion

These experiments demonstrate that these three simple but powerful behaviors are sufficient for a wide variety of quasi-static tasks, and even some dynamic tasks (e.g., berthing). Compared with more direct implementations of force control or teleoperation, PTWL was observed to be capable of executing a wide variety of manipulation tasks safely and robustly, both under supervisory control and within autonomous state machines. These techniques will allow robots to effectively achieve demanding mission goals for servicing, assembly and manufacturing in harsh environments and subject to communications delays and disruptions.

It is unknown what additional low-level, compliant-motion behaviors might be valuable for either supervisory control or autonomous operation involving manipulation tasks. At present, the three behaviors described here have been found to be remarkably competent and versatile.

## Data Availability

The datasets presented in this study can be found in online repositories. The names of the repository/repositories and accession number(s) can be found below: https://www.youtube.com/watch?v=ALYjr_WGz7M&list=PLxDJ3XzZwbw3KdwHAJfbJLMin-bL7g0VXl.
